# Optimization of Loading Path for Hydroforming of Asymmetric Curved Tubes Using AMGA

**DOI:** 10.3390/ma19143046

**Published:** 2026-07-15

**Authors:** Zaixiang Zheng, Hui Tan, Gang Wu, Feng Wang, Siyuan Tang, Yujie Chen, Yang Zhao, Hantao Yu, Zhengjian Pan

**Affiliations:** 1 School of Mechanical Engineering, Yangzhou University, Yangzhou 225012, China; 2 Yangzhou Hangying Technology Equipment Co., Ltd., Yangzhou 225800, China

**Keywords:** tube hydroforming, loading path, torsion beam trailing arm, AMGA, optimization

## Abstract

The hydroforming performance of trailing arms is governed by the coupled effects of feed parameters, pressure schedules and frictional characteristics. Improper parameter matching readily induces typical forming defects such as wrinkling, cracking and uneven wall thickness. To address this issue, a multi-objective optimization method for hydroforming is proposed in this study. Taking the maximum wall thickness, minimum wall thickness and die-to-workpiece gap of the tubular blank as optimization objectives, and the internal pressure and right-side axial feed velocity as design variables, an integrated numerical simulation framework combining the Archive-based Micro Genetic Algorithm (AMGA) and LS-DYNA is established to analyze the hydroforming process. By adaptively adjusting the key control points of internal pressure and axial feed loading curves, the developed method expands the solution space and realizes the automatic optimization of loading paths. The results reveal that the maximum wall thinning rate of the tubular component drops from 20.4% to 14.8%. Meanwhile, the wall thickness uniformity is improved and forming defects are effectively suppressed while the thickening rate remains stable. Furthermore, a complete round of optimization calculation involving thousands of finite element solutions can yield a complete set of Pareto non-dominated solutions. In this paper, the AMGA multi-objective optimization algorithm is adopted to acquire the optimal loading paths, and physical prototype experiments are carried out relying on self-developed 2000 T hydroforming equipment. Comparisons between measured and simulated wall thickness values of the tubular component show that the maximum relative error is controlled within 7.46%, which verifies the reliable engineering applicability of the proposed optimization scheme and provides new insight into the process optimization for forming similar structural components.

## 1. Introduction

Torsion beam trailing arms are classified as asymmetric bent tubular components with complex and variable cross-sectional geometries along the axial direction. The two ends of the component feature flat and elliptical cross-sections respectively, without transitional fillets, making such parts well-suited for fabrication via hydroforming [1,2]. The hydroforming process is synergistically governed by multiple process parameters, including internal forming pressure, axial feed displacement, friction coefficient and die corner radius. These parameters feature strong coupling and highly nonlinear interactions. Meanwhile, trade-off relationships commonly exist between different optimization objectives for forming performance [3,4,5]. Conventional single-objective optimization approaches and trial-and-error methods fail to determine the optimal combination of process parameters accurately. Accordingly, it is imperative to develop an efficient multi-parameter collaborative optimization strategy for hydroforming process optimization.

In recent years, researchers at home and abroad have carried out extensive systematic research on the multi-objective parameter optimization of tube hydroforming. Domestic scholars mostly employ intelligent optimization algorithms to match optimal loading paths and process parameters. Wang et al. [6] integrated finite element simulation and numerical optimization methods to solve the optimal loading curves for hydroformed T-shaped tubes, and verified the effectiveness of this optimization strategy via physical experiments. Targeting typical defects including excessive wall thinning and wrinkling occurring in the forming of double-convex tubes, Zhang et al. [7] quantitatively analyzed the influence laws of loading path parameters on forming quality by means of the response surface method. Taking automotive instrument panel beams as engineering research objects and setting wall thickness uniformity as the optimization index, Zheng et al. [8] adopted the NSGA-II algorithm to optimize core process parameters such as internal pressure and axial feed displacement, and obtained a process scheme with controllable forming defects. On this basis, Chang et al. [9] conducted parameter optimization for the preforming process of rectangular tubes and screened the optimal loading paths from the Pareto optimal solution set generated by NSGA-II. Xu et al. [10] introduced a fuzzy control algorithm to optimize the loading trajectories of variable-diameter tubes, which remarkably improves the wall thickness uniformity and overall forming quality of tubular parts. Based on finite element simulation, Zhang et al. [11] constructed a Kriging surrogate model to characterize the mapping relationship between process parameters and forming quality, and accomplished the collaborative optimization of multiple process parameters using the NSGA-III algorithm. Foreign relevant studies focus more on analyzing the plastic flow behaviors of materials combined with numerical algorithms. Raut et al. [12] coupled the material constitutive model and the ALE numerical technique to describe the material flow characteristics during the forming stage, and completed the hydroforming process optimization relying on design of experiments (DOE) and response surface methodology.

A comprehensive review of the aforementioned domestic and overseas research reveals that most existing multi-objective optimization studies on tube hydroforming are implemented based on finite element simulation platforms. The response surface method and NSGA-II series genetic algorithms are widely adopted to realize the collaborative matching of loading paths and process parameters, and some investigations further combine material constitutive equations and ALE numerical techniques to analyze the material flow laws during forming. Nevertheless, existing hydroforming optimization frameworks have notable limitations. Conventional multi-objective evolutionary algorithms generally suffer from high computational costs for iterative calculation and limited convergence speed. When calculations are performed with small populations, it is difficult to simultaneously guarantee the solution accuracy and distribution diversity of the Pareto solution set. Meanwhile, the majority of existing optimizations are carried out merely for single components and specific forming working conditions, leading to limited universality of the corresponding process regulation laws. Few studies deeply analyze complex plastic deformation mechanisms under coupled multi-field conditions [13]. To alleviate the above drawbacks of traditional optimization algorithms in the high-cost finite element coupled optimization scenarios of hydroforming, the Archive-based Micro Genetic Algorithm (AMGA) [14] is introduced in this paper to conduct global process optimization. The Archive-based Micro Genetic Algorithm streamlines computation using historical search information and produces high-quality non-dominated solutions efficiently with small populations. It outperforms traditional algorithms in complex multi-objective optimization, yet it has never been applied to tube hydroforming.

In this study, AMGA is coupled with LS-DYNA simulation. Taking wall thickness indicators and die-to-workpiece gap as objectives, and internal pressure and axial feed velocity as design variables, we realize global loading path optimization by regulating key control points of loading curves. The applicability of AMGA in hydroforming is validated to mitigate cracking, wrinkling and other defects, and to provide an efficient approach for complex plastic forming optimization.

## 2. Models, Equipment and Instruments

### 2.1. Structural Features of Trailing Arm

**Figure 1:**
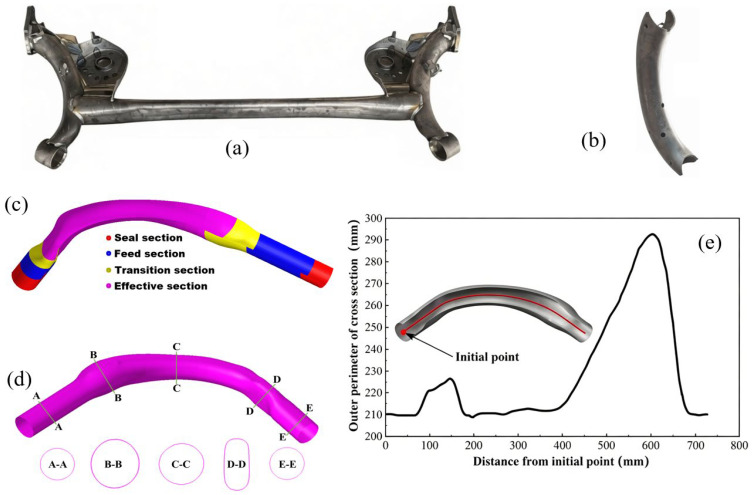
Trailing arm structural features: (**a**) torsion beam assembly, (**b**) trailing arm, (**c**) hydroforming die cavity, (**d**) cross-sectional shapes, (**e**) outer perimeter of cross section.

The torsion beam trailing arm is arranged along the longitudinal direction of the vehicle body. Its main body is welded to the torsion beam cross member for assembly integration. Its front end is hinged to the vehicle body via bushings, which provides longitudinal positioning and acts as a swing pivot., while the rear end is rigidly connected to the wheel bearing housing through a flange structure. This configuration enables effective transmission of driving force, braking force and longitudinal loads during vehicle operation [15,16]. The overall assembly structure is presented in Figure 1a. As shown in Figure 1b, the component is a complex hollow part with asymmetric geometry and large curvature. To guarantee forming stability during hydroforming, additional sealing sections, material feeding sections and transition sections are required, as illustrated in Figure 1c. This trailing arm exhibits dramatic variations in cross-sectional profiles along its axial direction, with flat and elliptical cross-sections at its two ends respectively (Figure 1d). The minimum perimeter of the cross section is 209.42 mm, corresponding to an equivalent diameter of 66.67 mm, and the maximum perimeter reaches 293.61 mm with an equivalent diameter of 93.51 mm. The relative difference between the two values is 40.18%. Such a large structural discrepancy from the initial tubular blank leads to considerable difficulties in the forming process (Figure 1e).

### 2.2. Equipment and Die Assembly

**Figure 2:**
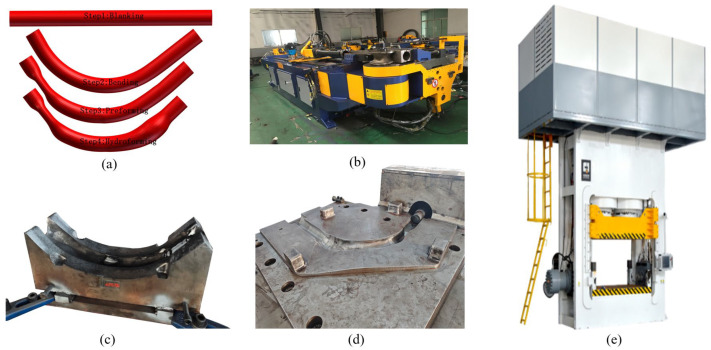
Equipment and die assembly: (**a**) forming process of trailing arm, (**b**) initial tubular blank, (**c**) preforming die, (**d**) hydroforming die, (**e**) 2000-ton hydraulic forming press.

The manufacturing of asymmetric bent tubular components consists of multiple procedures including pre-bending, preforming and hydroforming. Multi-stage incremental forming technology acts as a critical technical route for the precision fabrication of complex hollow tubular parts [17,18]. Since the central axis of the torsion beam trailing arm presents an irregular curved profile, the initial straight tubular blank cannot fit the cavity of hydroforming dies directly. For this reason, pre-bending and preforming are adopted as preliminary processes for blank pretreatment, as shown in Figure 2a. Firstly, the initial straight blank is subjected to axial pre-bending using pipe bending equipment (Figure 2b). Afterwards, the pre-bent blank is placed into the preforming die for stamping and shaping (Figure 2c). After pretreatment, the tube’s axial profile approximately matches the target die cavity contour (Figure 2d), which enables reliable assembly and positioning inside the dies. Ultimately, the integral precision forming of the complex trailing arm component is realized using a self-developed 2000-ton hydroforming press (Figure 2e).

### 2.3. Material Property Tests and Material Constitutive Model

**Figure 3:**
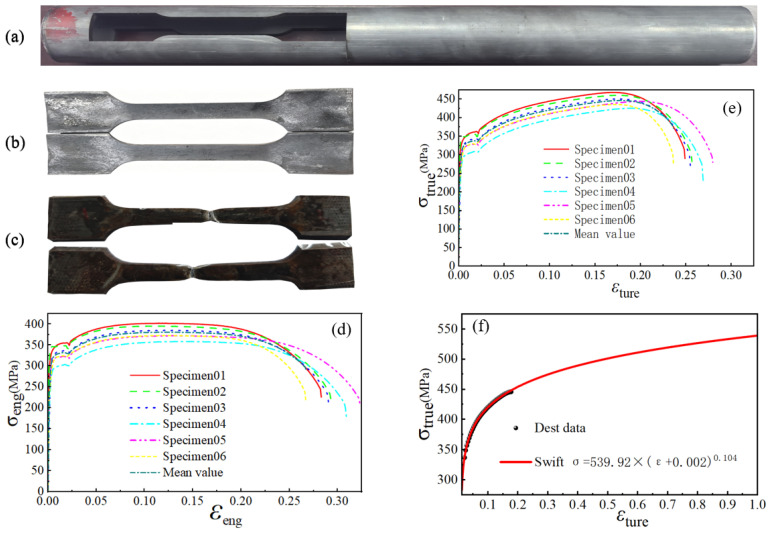
Material testing and data processing: (**a**) tube, (**b**) standard specimen, (**c**) tested specimens, (**d**) engineering stress–strain curve, (**e**) true stress–strain curve, (**f**) fitted true stress–strain curve.

The material constitutive model can accurately describe the intrinsic relationship between stress and strain, and acts as a fundamental mechanical basis for guaranteeing the calculation accuracy of finite element numerical simulation in plastic forming [19]. In this work, six groups of standard tensile specimens were machined from the initial tubular blank (Figure 3a). Uniaxial tensile tests were then conducted using an electronic universal testing machine, which was manufactured by Sansi Zongheng, Shanghai, China, as presented in Figure 3c, to acquire the engineering stress–strain data of the material (Figure 3d). Since engineering stress and strain are calculated based on the original dimensions of specimens, they fail to account for cross-sectional contraction and axial elongation induced by plastic deformation. Accordingly, such data cannot precisely reflect the actual mechanical response during the necking stage [20,21]. For this reason, the engineering stress–strain curve is converted into the true stress–strain curve via theoretical formulas, so as to provide accurate material parameters for subsequent numerical simulation (Figure 3e). The specific conversion formulas are given as follows:

(1) $$\sigma_{\mathrm{ture}}=\sigma_{\mathrm{eng}}\times \left(1+\varepsilon_{\mathrm{eng}}\right)$$

(2) $$\varepsilon_{\mathrm{ture}}=\ln\left(1+\varepsilon_{\mathrm{eng}}\right)$$

where: $\sigma_{\mathrm{eng}}$: engineering stress (MPa); $\varepsilon_{\mathrm{eng}}$: engineering strain; $\sigma_{\mathrm{ture}}$: true stress (MPa); $\varepsilon_{\mathrm{ture}}$: true strain.

To eliminate the distortion of experimental data caused by necking effect, all measured data were truncated at the uniform plastic deformation stage. On this basis, the Swift hardening model was adopted to fit the true stress–strain curves. The fitting results are presented in Figure 3f, which can provide accurate input parameters for the material constitutive model in subsequent finite element simulations.

(3) $$\sigma =a\times {\left(\varepsilon +b\right)}^{n}$$

where: σ: Stress (MPa); ε: Strain.

## 3. Hydroforming: Principle and Failure Modes

### 3.1. Hydroforming Principle

**Figure 4:**
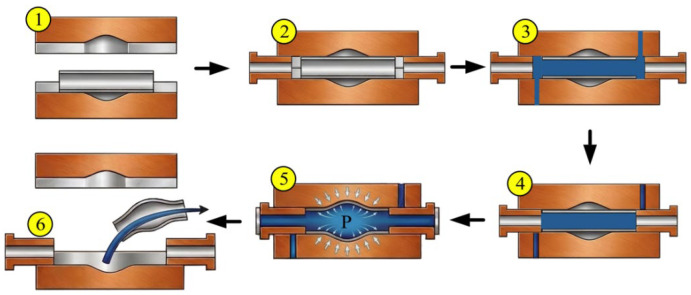
Schematic of tube hydroforming principle.

Tube hydroforming is an advanced plastic forming process derived from traditional hydraulic bulging [22]. In this process, metal tubes are used as blanks. High-pressure fluid is injected into the sealed tube cavity, and axial thrust is applied at both ends of the tube blank for material feeding. Under the combined action of internal pressure and axial load, the tube blank undergoes plastic deformation and gradually adheres to the die cavity, ultimately forming high-precision hollow structural components [23,24,25]. The forming principle is illustrated in Figure 4.

### 3.2. Trailing Arm Forming Process

The overall forming process system for torsion beam trailing arms is relatively complex, which consists of multiple critical procedures including blanking, pre-bending, preforming, annealing treatment, hydroforming, laser trimming and punching [26]. Firstly, high-frequency longitudinal welded steel tubes are cut to specified lengths. The pipe blank is shaped by bending equipment to make its axial profile close to the target configuration. Subsequent preforming and shaping are performed to meet the assembly requirements of hydroforming dies. After annealing, the blank is subjected to hydraulic bulging. Finally, laser cutting is adopted for edge trimming and punching finishing, realizing the integrated manufacturing of finished components.

### 3.3. Hydroforming Failure Modes

**Figure 5:**
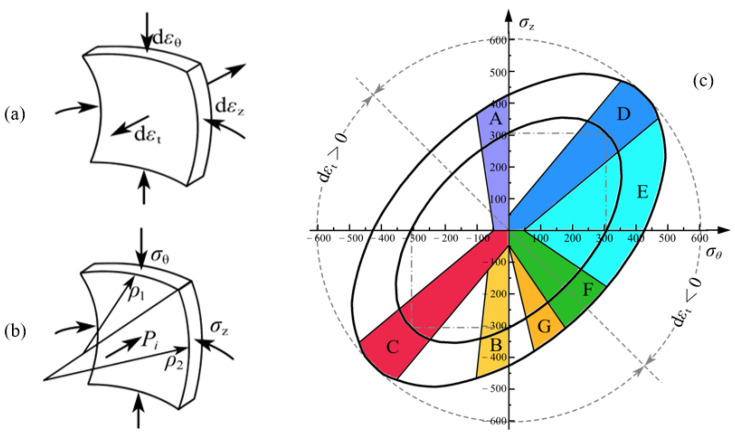
Stress and strain state of micro-element: (**a**) strain condition, (**b**) stress condition, (**c**) distribution range of micro-element on the yield locus under plane stress.

Typical forming defects including wrinkling, folding, severe wall thinning and cracking mainly occur in the hydroforming bulging zone of trailing arms [27]. To clarify the failure mechanism in this forming process, it is essential to analyze the stress and strain characteristics. Assuming that the deformation of tube elements complies with the thin-shell theory, an infinitesimal element is selected arbitrarily within the bulging zone. Its stress state and strain distribution are presented in Figure 5a,b, respectively.

According to the volume constancy principle, the strains of the element in three directions satisfy the following equation:

(4) $$\varepsilon_{\theta }+\varepsilon_{z}+\varepsilon_{t}=0$$

where: $\varepsilon_{\theta }$: Circumferential strain of the element; $\varepsilon_{z}$: Axial strain of the element; $\varepsilon_{t}$: Thickness strain of the element.

Stress analysis is conducted on the infinitesimal element in Figure 3b, and the corresponding governing equations are expressed as:

(5) $$\frac{\sigma_{\theta }}{\rho_{1}}+\frac{\sigma_{z}}{\rho_{2}}=\frac{P_{i}}{T_{i}}$$

where: $\sigma_{\theta }$: Circumferential stress of the element (MPa); $\sigma_{z}$: Axial stress of the element (MPa); $\rho_{1}$: Minor radius of curvature of the element (mm); $\rho_{2}$: Major radius of curvature of the element (mm).

The distribution range of the element on the yield locus under plane stress is shown in Figure 5c. The equivalent stress and equivalent strain under the plane stress condition are formulated as:

(6) $$\bar{\sigma }={\left[1-\frac{\sigma_{z}}{\sigma_{\theta }}+{\left(\frac{\sigma_{z}}{\sigma_{\theta }}\right)}^{2}\right]}^{1/2}\cdot \sigma_{\theta }$$

(7) $$\bar{\varepsilon }={\left\{\frac{4}{3}\left[1+\frac{\varepsilon_{z}}{\varepsilon_{\theta }}+{\left(\frac{\varepsilon_{z}}{\varepsilon_{\theta }}\right)}^{2}\right]\right\}}^{1/2}\cdot \varepsilon_{\theta }$$

where: $\bar{\sigma }$: Equivalent stress of the element (MPa); $\bar{\varepsilon }$: Equivalent strain of the element.

**Figure 6:**
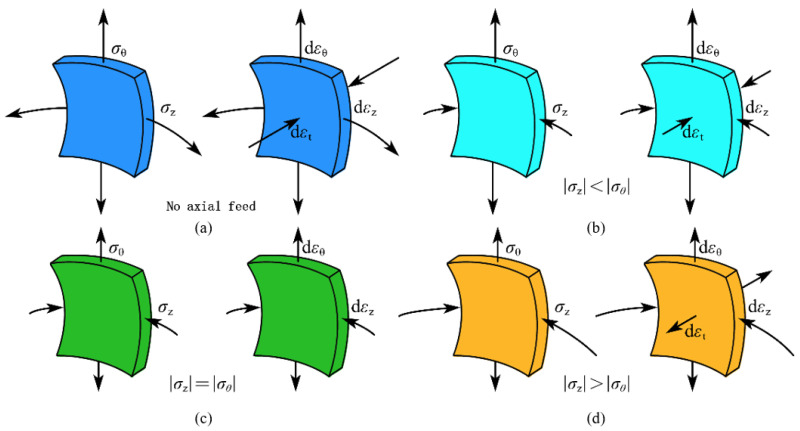
Stress and plastic deformation state of micro-element during hydroforming: (**a**) no axial feed, (**b**) insufficient axial compression, (**c**) balanced axial–circumferential stress, (**d**) excessive axial material feeding.

In tube hydroforming, plastic deformation mainly occurs in the free bulging zone where the tube blank is not in contact with the die cavity. Under the coordinated regulation of forming internal pressure and axial feed, the material in this zone exhibits four typical plastic strain states, which correspond to four forming conditions: no axial feed, insufficient axial compression, balanced axial and circumferential stress, and excessive material feeding [28,29]. The corresponding strain mechanisms are illustrated in Figure 6. During the pressure rising and calibration stages with no axial feed, the tube blank is subjected to biaxial tensile stress. Both axial and circumferential elongation take place simultaneously, leading to remarkable wall thinning, which corresponds to the stress state of Region D in Figure 5. When the axial compressive stress is inadequate to counteract the wall thinning induced by circumferential tension, the material still suffers slight thickness reduction, as represented by Region E in the figure. When a dynamic balance is achieved between axial material feeding and internal pressure, the axial compressive strain effectively compensates for wall thickness loss caused by circumferential tension, thereby realizing circumferential expansion with a constant wall thickness. This condition matches the stress state of Region F. By contrast, excessive axial material feeding results in redundant axial compressive deformation that cannot be converted into circumferential material flow. This phenomenon easily causes material accumulation and wall thickening, and further triggers wrinkling defects, corresponding to the stress state of Region G.

## 4. Models, Methods and Experiments

### 4.1. Finite Element Modeling and Methods

#### 4.1.1. Numerical Simulation Model for Hydroforming Analysis

**Figure 7:**
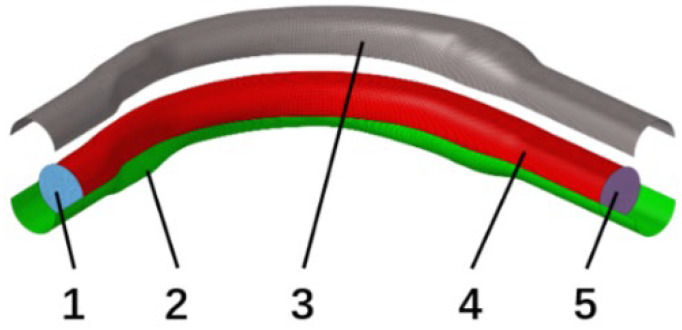
Finite element model: 1. Left plug, 2. Lower die, 3. Upper die, 4. Tube blank, 5 Right plug.

In this work, SPHC steel tubes with an outer diameter of 67 mm and initial wall thickness of 5.0 mm are used as forming blanks. Taking multiple process constraints including part geometric dimensions, axial feed stroke, end sealing transition structure and post-machining cutting margin into consideration, the initial length of the tubular blank is finally determined as 958 mm. The detailed finite element modeling settings are presented as follows. The die cavity and end plugs are defined as rigid bodies meshed with BT shell elements using single-point in-plane integration. The deformable tube blank also uses BT shell elements with full in-plane integration, with five integration points defined through the thickness. A combined stiffness–viscosity hourglass control algorithm is adopted to stabilize numerical calculations, with the hourglass stiffness coefficient set to 0.05. All energy terms including hourglass energy, friction energy, shell membrane bending shear energy and damping energy are activated for output monitoring. A symmetric penalty contact algorithm governs all contact pairs, and the Coulomb friction model describes interfacial friction with a coefficient of 0.05. The basic material physical parameters are determined according to the national standard of SPHC steel and the tube hydroforming simulation conditions. Based on SPHC steel national standard mechanical properties and tube hydroforming simulation requirements, the material’s basic physical parameters are defined as: elastic modulus E = 205 GPa, Poisson’s ratio ν=0.29, material density ρ = 7830 kg/m^3^. Constitutive parameters were obtained from the experimental fitting results shown in Figure 3f, and the constitutive equation is: $\bar{\sigma }=539.92\times {\left(0.002+\bar{\varepsilon }\right)}^{0.104}$ [30], where $\bar{\sigma }$ stands for true stress and $\bar{\varepsilon }$ for true strain. Figure 7 shows the developed finite element model.

#### 4.1.2. Selection of Numerical Simulation Algorithms

The existing nonlinear mechanical analytical framework for tubular components can accurately characterize cross-sectional distortion induced by large displacements and large rotations, with a relatively complete theoretical system [31]. However, these analytical methods are only valid for linear elastic states and have severe practical limitations, as they cannot capture the core physical mechanisms of hydroforming. The practical hydroforming process of torsion beam trailing arms involves multiple strongly nonlinear physical effects including plastic strain evolution, material work hardening, dynamic interfacial contact friction, cumulative preforming strain over multiple forming stages, and stress relief after annealing. Conventional purely elastic analytical equations cannot precisely describe the full-process evolution laws under multi-field coupling, and thus fail to meet the high-precision analysis requirements of this work.

To address the above deficiencies of theoretical analytical methods, the LS-DYNA numerical simulation method is selected as the core solving approach in this paper. Compared with the traditional static implicit algorithm, the implicit algorithm requires iterative solving of the global stiffness matrix at every load increment. The studied forming process includes mold closing, axial feeding and pressure boosting, with frequent abrupt contact state switches between tube and die, which readily causes iterative non-convergence. The dynamic explicit algorithm adopts the central difference explicit time integration scheme to solve the governing dynamic equations step by step. It avoids assembling and iteratively solving the global stiffness matrix, thus greatly reducing convergence issues typical of strongly nonlinear simulations [32]. This algorithm exhibits superior adaptability to strongly nonlinear conditions featuring large plastic deformation and variable contact states with better solving stability. It can fully reproduce the deformation characteristics coupled by geometric, material and contact nonlinearities throughout the entire hydroforming process of torsion beam trailing arms, accurately capture the evolution of stress and strain, generate forming defects and stress relief laws in each working stage, and is perfectly suited for the full-process numerical simulation of torsion beam hydroforming in this research. The 25 ms virtual calculation time is achieved via mass scaling time compression, instead of representing the real physical forming duration; this approach drastically cuts the computational cost of iterative optimization. To satisfy the quasi-static assumption, the ratio of kinetic energy to internal energy is monitored throughout the simulation, with a threshold value of less than 5% adopted as the verification criterion. This criterion is satisfied at all time steps of the model, indicating negligible inertial effects and valid, reliable simulation results.

#### 4.1.3. Loading Path Design

**Figure 8:**
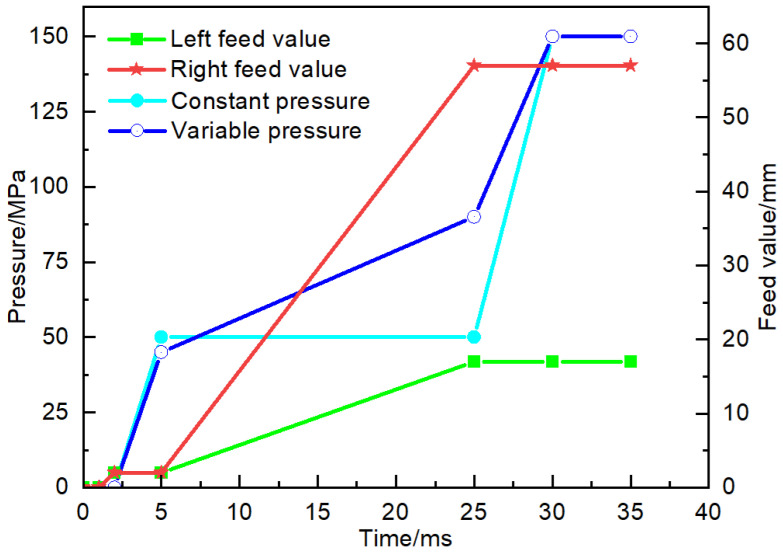
Axial feed and internal pressure loading curves.

The internal pressure loading curve for hydroforming can be divided into four characteristic stages: initial yielding, forming, calibration and pressure holding. Among them, the forming and calibration stages play a decisive role in the forming quality of components. A low-speed pressure rise mode is adopted in the forming stage to ensure stable plastic deformation of the tube blank. In the calibration stage, the pressure rises rapidly, which not only accurately corrects the profile and dimension of components but also shortens the forming cycle and improves processing efficiency. The axial feed displacement curve is dynamically regulated according to forming characteristics. Rapid material feeding is implemented during the forming stage to meet the bulging requirement. Since the blank undergoes negligible deformation in the calibration stage, axial material feeding is basically terminated. Considering the equipment operating conditions, mechanical properties of the material and coupling rules of processes, two internal pressure loading schemes are proposed in this study, namely constant-pressure feeding mode and pressure-rise feeding mode. A comparison of the loading curves is presented in Figure 8.

#### 4.1.4. Evaluate the Effect of Element Size on Forming Results

**Figure 9:**
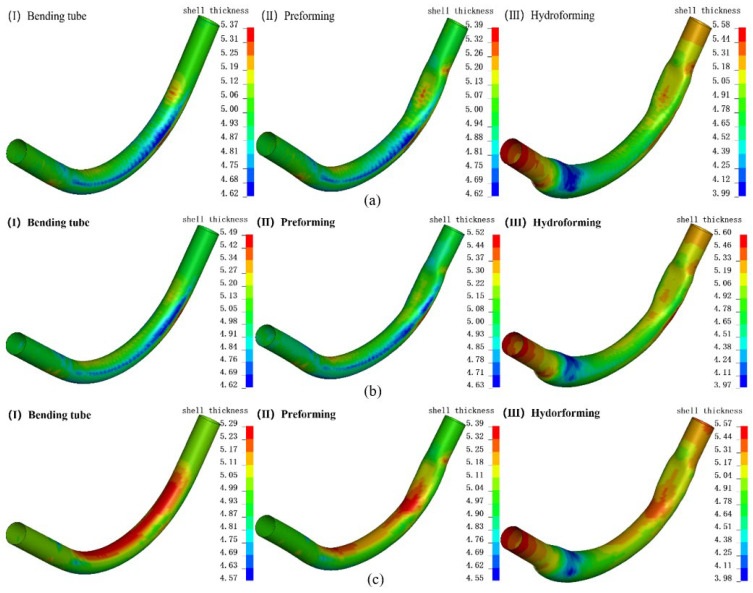
Wall thickness contours under different mesh sizes: (**a**) 2 mm, (**b**) 4 mm, (**c**) 6 mm.

Mesh element size is a key parameter affecting the accuracy and computational efficiency of finite element simulation. To balance simulation accuracy and the computational efficiency of subsequent multi-objective optimization, a mesh size sensitivity analysis is necessary. Three element sizes of 2 mm, 4 mm and 6 mm were adopted for comparative simulations in this study. The detailed parameters are listed in Table 1, and the wall thickness distribution contours of the component under different mesh schemes are presented in Figure 9.

As can be seen from the simulation time consumption results listed in Table 1, mesh size exerts a remarkable influence on the computational efficiency of numerical simulation. The 2 mm mesh delivers the highest calculation accuracy, yet a single full simulation takes 74 min and 35 s. Running all 1600 simulation evaluations would take 82 days and 20 h, not counting extra time for post-processing and algorithm iteration. Such excessive computational cost renders this mesh impractical for engineering applications. By contrast, one full forming simulation with the 6 mm mesh only costs 1 min and 6 s, and the total time for the complete set of 1600 simulations is 37 h, 28 min and 57 s, representing a controllable computation period. Mesh convergence analysis reveals that the relative calculation error of wall thickness for all three mesh schemes is less than 1%. Balancing numerical convergence accuracy and the computational overhead of large-scale iterative calculations, a uniform element size of 6 mm is adopted for all optimization simulations in this work.

#### 4.1.5. Experimental Verification

**Figure 10:**
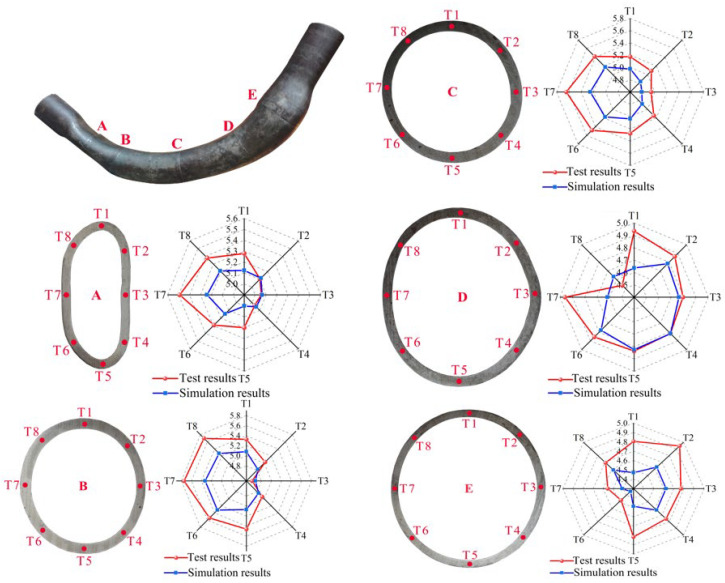
Thickness distribution of different section characteristics.

To verify the reliability of the simulated loading paths and the formability of the tube hydroforming process, process experiments were carried out on a self-developed 2000-ton hydroforming apparatus following the conventional loading paths. After integrated processing including tube bending, preforming, annealing and hydroforming, the fabricated specimens exhibited sound forming quality without typical defects such as cracking and wrinkling. Wall thickness comparison was performed at eight characteristic points on five critical cross-sections of the component, as shown in Figure 10. The experimental and numerical results are listed in Table 2. The results reveal that the maximum wall thickness of the component is 5.75 mm with a thickening rate of 15%, and the minimum wall thickness is 4.18 mm with a thinning rate of 16.4%. The wall thickness deviation between experimental measurements and simulation predictions is less than 8% at all detection points, with the maximum deviation of only 7.46%. The two sets of data show good agreement, which effectively validates the accuracy of the established simulation model.

### 4.2. Optimization Models and Methods

#### 4.2.1. Determination of Design Variables

The trailing arm of the torsion beam features obvious variations in cross-sectional dimensions. Its equivalent diameter at the middle section is 93.8 mm, while the diameter at the end section is only 67 mm. During the pressure rising stage of hydroforming, axial loads are applied to the ends of the tube blank synchronously. This measure overcomes the interfacial frictional resistance and drives material at the ends to flow toward the middle region, which effectively restrains excessive local wall thinning and cracking. Meanwhile, it ensures tight contact between the feeding head and the inner wall of the tube blank, so as to maintain the sealing performance of the forming cavity. It is thus clear that the parameter matching between internal pressure and axial feed loading curves is critical to forming quality. To address the problem of automatic iterative regulation of loading paths in the integrated optimization process, a parametric modeling method based on control points is adopted in this study. The coordinates of key control points on the curves are defined as design variables. The adaptive adjustment of pressure and feed paths is realized by iteratively updating the parameters of control points, which is expressed as:

(8) $$P(t)=\left[P_{1},P_{2},\ldots ,P_{m}\left|t_{1},t_{2},\ldots ,\right.t_{m}\right]$$

(9) $$V(t)=\left[V_{1},V_{2},\ldots ,V_{n}\left|t_{1},t_{2},\ldots ,t_{n}\right.\right]$$

where: $P_{1},P_{2},\ldots ,P_{m}$ denotes the control point on the internal pressure loading curve; $V_{1},V_{2},\ldots ,V_{n}$ represents the control point on the axial feed velocity curve; while $t_{m}$ and $t_{n}$ are the corresponding time values for the pressure and velocity control points, respectively.

**Figure 11:**
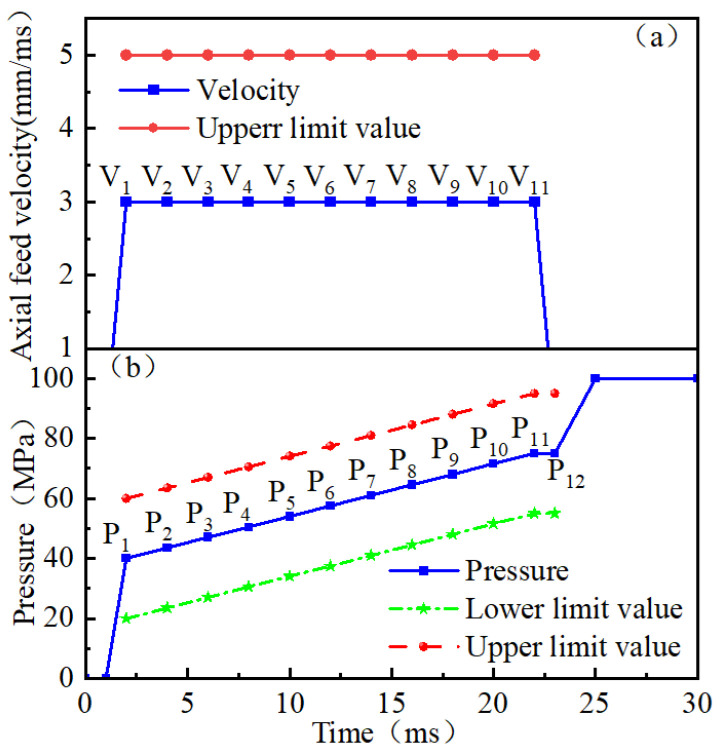
Internal pressure and axial feed velocity design variables: (**a**) internal pressure control points and their boundaries, (**b**) feed velocity control points and their boundaries.

Combined with the structural characteristics of the part and forming process requirements, 12 control points are selected on the pressure curve and 11 on the velocity curve. A total of 23 control points are defined as design variables, and the detailed curves are presented in Figure 11.

#### 4.2.2. Determination of Objective Functions

**Figure 12:**
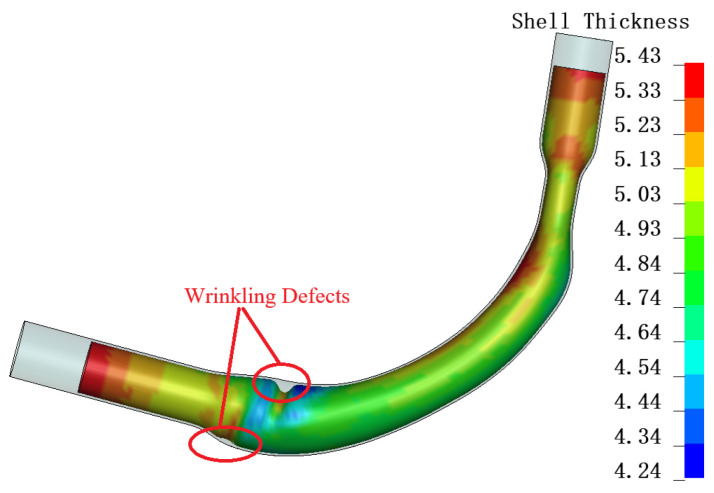
Wrinkling defects in tube hydroforming.

Compared with straight symmetric tubular parts, the curved-axis trailing arm suffers from severely uneven material flow during hydroforming, which readily induces forming defects such as unilateral wrinkling and fracture, as shown in Figure 12. Optimizing merely for the maximum and minimum wall thickness can suppress wrinkling and cracking, yet fails to guarantee the profile accuracy of the component. Accordingly, the maximum die fitting gap $S_{max}$ is introduced as the third objective function in this study to quantify forming accuracy and also evaluate the degree of wrinkling. The mathematical expressions of the three objective functions are presented as follows:

(10) $$f\left(p\left(t\right),v\left(t\right)\right)=\min\left(t_{max}\right)$$

(11) $$f\left(p\left(t\right),v\left(t\right)\right)=m\mathrm{ax}\left(t_{min}\right)$$

(12) $$f\left(p\left(t\right),v\left(t\right)\right)=m\mathrm{in}\left(S_{max}\right)$$

(13) $$S=(x_{s}-x_{p})\cdot n-\frac{1}{2}(t_{j}-t_{die})$$

where: $t_{\min}={\left\{t_{1},t_{2},\ldots ,t_{N1}\right\}}_{\min}$; $t_{\max}={\left\{t_{1},t_{2},\ldots ,t_{N1}\right\}}_{\max}$; $S_{\max}={\left\{S_{1},S_{2},\ldots ,S_{N2}\right\}}_{\max}$; $S_{j}$: Normal distance from the j-th node on the trailing arm to the die surface; $t_{i}$: Thickness of the i-th element after forming; $x_{S}$: Coordinate of the j-th node after forming; $x_{p}$: Coordinate of the nearest projection point of the j-th node on the die element face after forming; n: Unit outward normal vector of the die element face; N1: Total number of elements; N2: Total number of nodes.

#### 4.2.3. Selection of Multi-Objective Optimization Method

**Figure 13:**
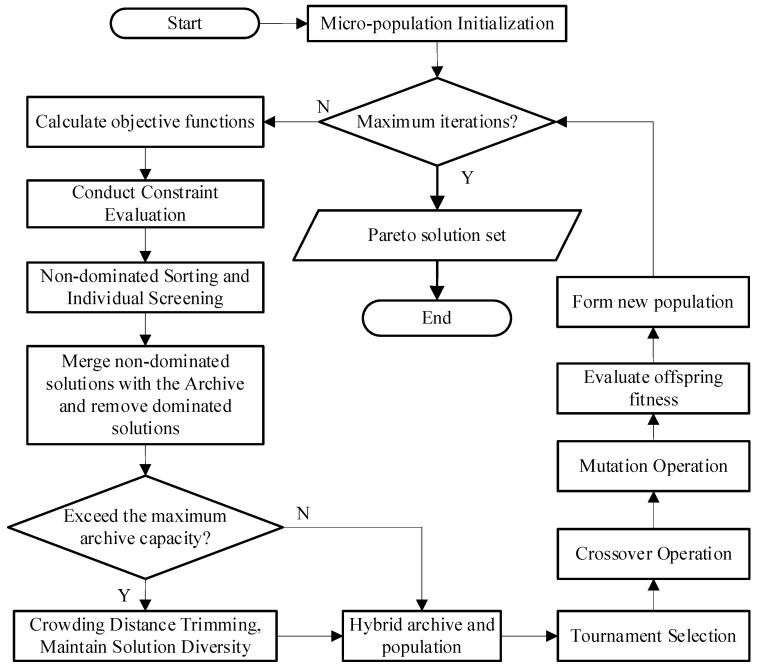
Flowchart of the AMGA.

Common multi-objective optimization algorithms include AMGA, NCGA, MOPSO and NSGA-II. As an improved multi-objective evolutionary algorithm, AMGA filters non-dominated solutions using a small working population and an external archive strategy. It features fast convergence, low computational cost and good diversity of Pareto solutions. Therefore, this algorithm is well suited for the time-consuming and highly constrained hydroforming process optimization in this work. The flowchart of AMGA is illustrated in Figure 13.

#### 4.2.4. Multi-Objective Optimization Process

**Figure 14:**
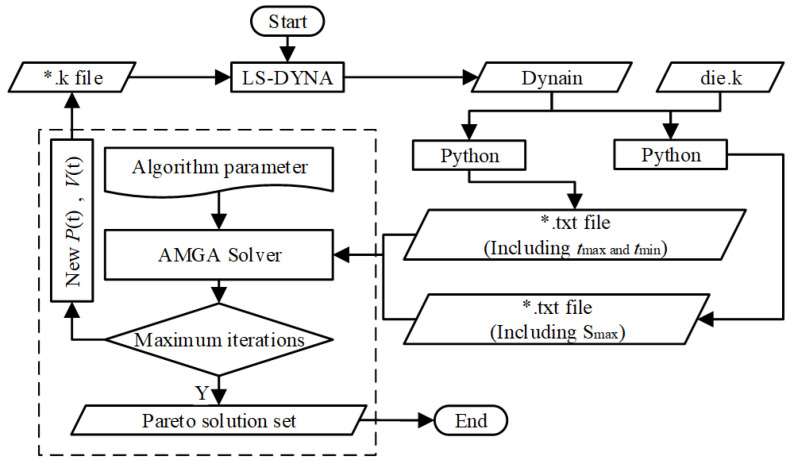
Multiple-objective optimization Flowchart.

An automated integrated multi-objective optimization system combining LS-DYNA and Python 3.11.4 secondary development is established in this study, and the overall iterative workflow is presented in Figure 14. Firstly, LS-DYNA reads the K file containing design variables, material parameters, die models and preforming results, conducts the hydroforming simulation, and outputs the dynain result file. Simulation data are subsequently extracted via Python scripts to calculate the maximum wall thickness, minimum wall thickness of the component and the maximum fitting gap between the component and die, followed by data export. Afterwards, the AMGA solver imports the target parameters to compute the population fitness, screen non-dominated solutions and update the external archive. Redundant individuals are eliminated based on the crowding distance mechanism to maintain the diversity of solution sets. The design variables are continuously updated throughout iterations, and the simulation loop repeats until the maximum iteration number is reached. Finally, the optimal Pareto solution set is obtained and output.

## 5. Results Evaluation

### 5.1. Simulation Results

**Figure 15:**
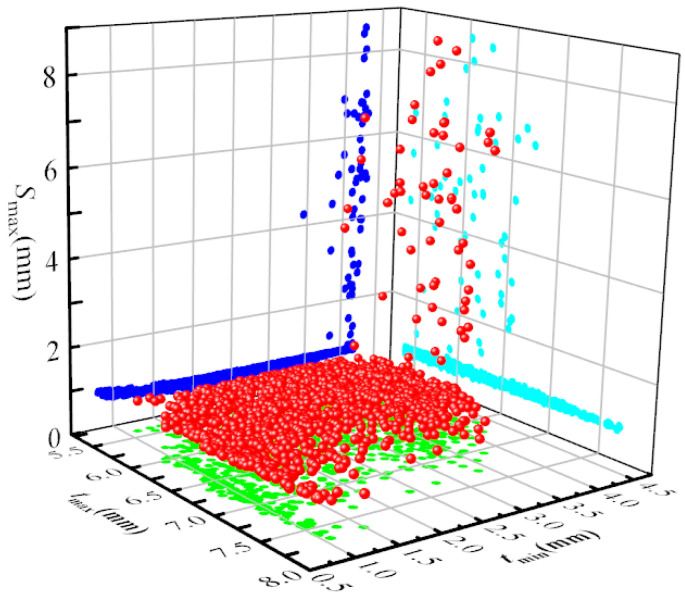
Scatter diagram of Pareto optimal solutions.

The key parameters of the Archive Micro-Genetic Algorithm (AMGA) were configured in this study. Both the initial population size and evolutionary population size were set to 160. The upper limits of the global archive and non-dominated solution set were defined as 500 and 200, respectively. The crossover probability and mutation probability were specified as 0.9 and 0.5, with corresponding distribution indices of 10 and 20. The maximum number of simulation evaluations was limited to 1600. The calculation results are presented in Figure 15. Each data point in the figure represents a feasible solution set. A smaller maximum wall thickness $t_{\max}$, smaller maximum fitting clearance $S_{\max}$ and larger minimum wall thickness $t_{\min}$ can substantially improve the forming quality and weight reduction performance of the tubular component. Accordingly, the solution sets located near the inner corner of Figure 15 are identified as the optimal solutions for this multi-objective optimization problem.

**Figure 16:**
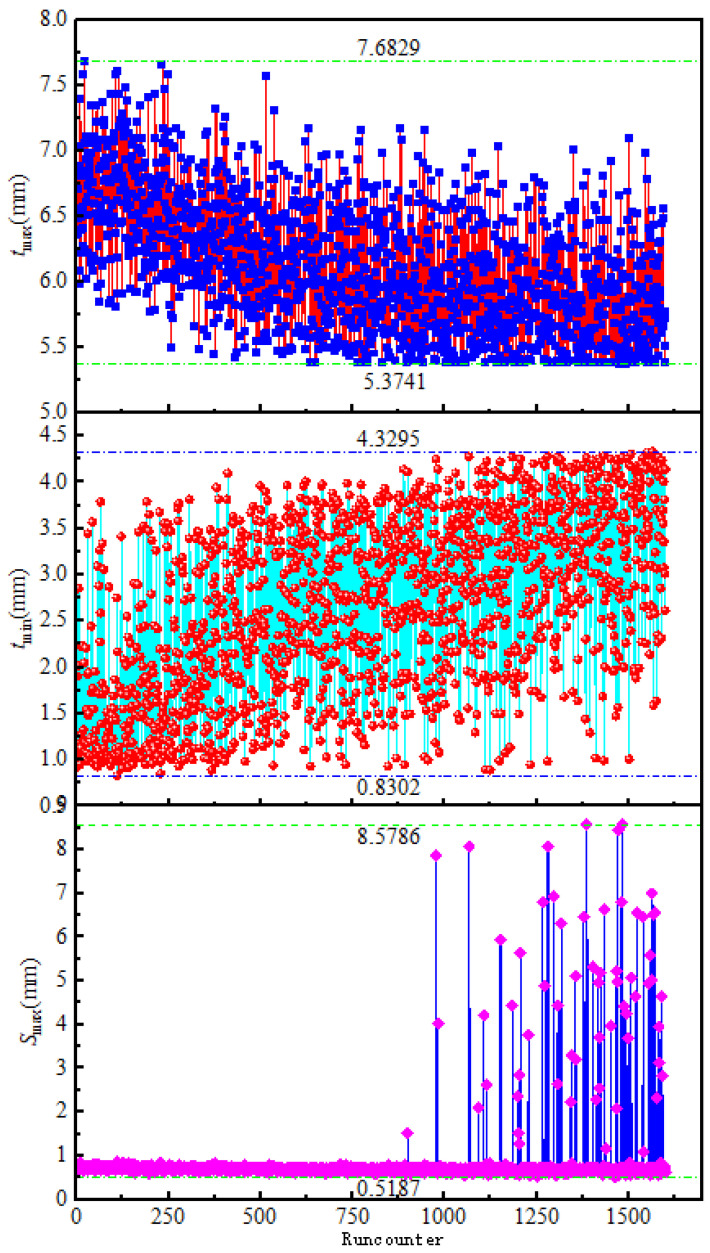
Variation in maximum and minimum wall thickness with iterations.

Figure 16 presents the simulation results, where the horizontal axis denotes the iteration number, and the vertical axes represent the maximum wall thickness $t_{\max}$, minimum wall thickness $t_{\min}$, and normal clearance $S_{\max}$ between the tube blank and die. The results show that $t_{\max}$ ranges from 5.37 mm to 7.68 mm, with the corresponding thickening rates of 7.4% and 53.6% respectively. Severe wrinkling occurs when the thickening rate reaches 53.6%. The values of $t_{\min}$ vary between 0.83 mm and 4.33 mm, leading to thinning rates of 83.4% and 13.4%. An extremely high thinning rate of 83.4% causes excessive local thinning and even cracking of the tube blank. In addition, $S_{\max}$ is measured from 0.52 mm to 8.56 mm. A clearance of 0.52 mm indicates an excellent contact condition between the trailing arm and die, and full fitting can be achieved by moderately increasing the forming pressure. By contrast, the maximum clearance of 8.56 mm induces prominent wrinkling and results in forming failure. Overall, the loading paths of internal pressure and axial feed are the dominant factors governing the forming quality and forming reliability of the tubular component.

**Figure 17:**
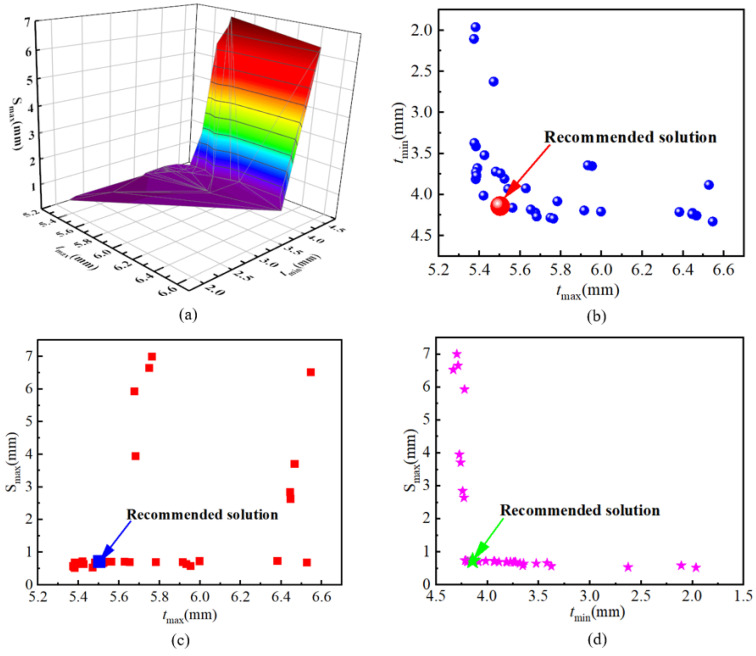
Three-objective Pareto optimal front and its two-dimensional projections: (**a**) 3D spatial surface of three-objective Pareto optimal front, (**b**) two-dimensional projection of Pareto solutions on the $t_{\max}$–$t_{\min}$ plane, (**c**) two-dimensional projection of Pareto solutions on the $t_{\max}$–$S_{\max}$ plane, (**d**) two-dimensional projection of Pareto solutions on the $t_{\min}$–$S_{\max}$.

Figure 17 shows the three-objective Pareto optimal front obtained via the AMGA together with its two-dimensional projection. The optimal solution set converges uniformly onto a continuous and irregular spatial surface, which exhibits good integrity without obvious vacancies or inferior scattered solutions. This verifies the excellent convergence and global optimization capability of the proposed algorithm. The analysis reveals that both $t_{\max}$ and $S_{\max}$ increase with the rise of $t_{\min}$. Nevertheless, no monotonic correlation exists between $t_{\max}$ and $S_{\max}$, and $S_{\max}$ presents remarkable uncertainty within a certain range. In addition, even when $S_{\max}$ reaches its optimal value, cracking may still occur if the minimum wall thickness is less than 3.0 mm. In contrast, an excessively large $S_{\max}$ will trigger severe wrinkling and eventually lead to forming failure. Therefore, it is essential to accurately evaluate the influence of $S_{\max}$ on the matching performance between process parameters and equipment machining conditions.

**Figure 18:**
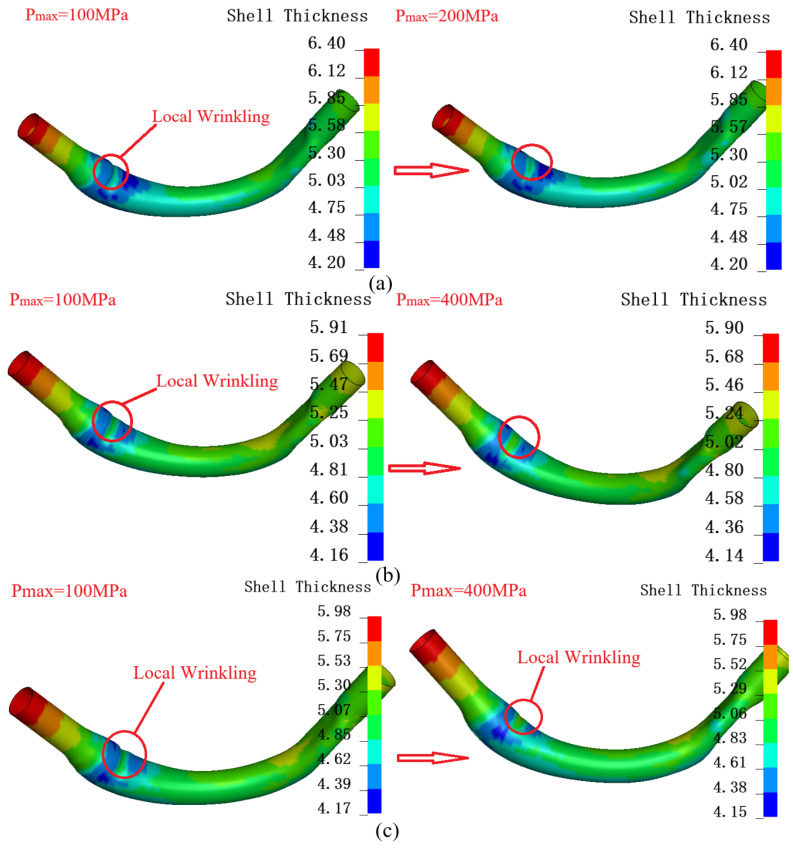
Pareto solutions under different maximum gap conditions: (**a**) S_max_ = 0.73 mm, (**b**) S_max_ = 1.09 mm, (**c**) S_max_ = 2.0 mm.

To further verify the technical feasibility of the optimal solutions, three Pareto optimal solutions with $S_{\max}$ of 0.73 mm, 1.09 mm and 2.0 mm were selected for evaluation, and the corresponding results are presented in Figure 18. The analysis indicates that the forming pressure required to eliminate die surface clearance rises remarkably with the increase of $S_{\max}$. As $S_{\max}$ increases from 0.73 mm to 1.09 mm, the maximum required forming pressure grows from 200 MPa to 400 MPa, representing a 100% increment. Moreover, the relatively large clearance cannot be fully eliminated even under a forming pressure of 400 MPa. This reveals that several theoretical Pareto optimal solutions fail to satisfy practical process constraints and carry risks of engineering failure. Meanwhile, the selection mechanism of the AMGA may also exclude some technically feasible solutions. Thus, further screening and verification are urgently required.

**Figure 19:**
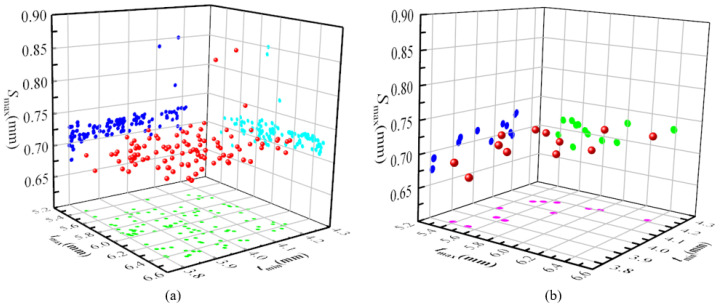
Comparison of all feasible solutions and feasible solutions in Pareto set: (**a**) all feasible process solutions of simulation results, (**b**) feasible process solutions in Pareto optimal solutions.

Given the mismatch between the aforementioned theoretical optimal solutions and practical manufacturing processes, a secondary screening was performed on the 1598 solution sets output by the AMGA, including two invalid solutions and all Pareto optimal solutions. Combined with the plastic forming characteristics of materials, equipment operating limits, practical production experience and simulation rules, the constraint thresholds for forming evaluation were defined as follows: the upper limit of: $t_{\max}$ was 6.0 mm, the upper limit of $S_{\max}$ was 1.0 mm, and the lower limit of $t_{\min}$ was 3.8 mm. The screening results are illustrated in Figure 19, where all scattered points represent process-feasible solutions. Comparative analysis shows that the number of feasible solutions within the Pareto optimal set is obviously lower than that of all practical feasible solutions, which is attributed to the inherent screening mechanism of the AMGA. This algorithm only retains optimal solutions that satisfy the non-dominated relationship, uniform distribution and hard constraints, and eliminates redundant solutions via multiple procedures such as archive screening, crowding distance trimming, evolutionary selection and constraint judgment. To maintain the diversity of solution sets and avoid aggregation on the Pareto front, densely distributed non-dominated feasible solutions are trimmed, while dominated feasible solutions with low crowding degree are directly discarded. In conclusion, the loss of partial feasible solutions is an inevitable outcome of the multi-stage screening and optimization of the algorithm, which is a normal convergence characteristic of multi-objective optimization.

### 5.2. Re-Optimization of Results

The aforementioned unconstrained simulation optimization was performed to expand the solution space and obtain abundant Pareto optimal solutions, so as to provide sufficient alternatives for forming process planning and process parameter matching. Nevertheless, the solution set acquired under this mode features a wide coverage and a large quantity, containing numerous invalid solutions that violate practical engineering constraints.

**Figure 20:**
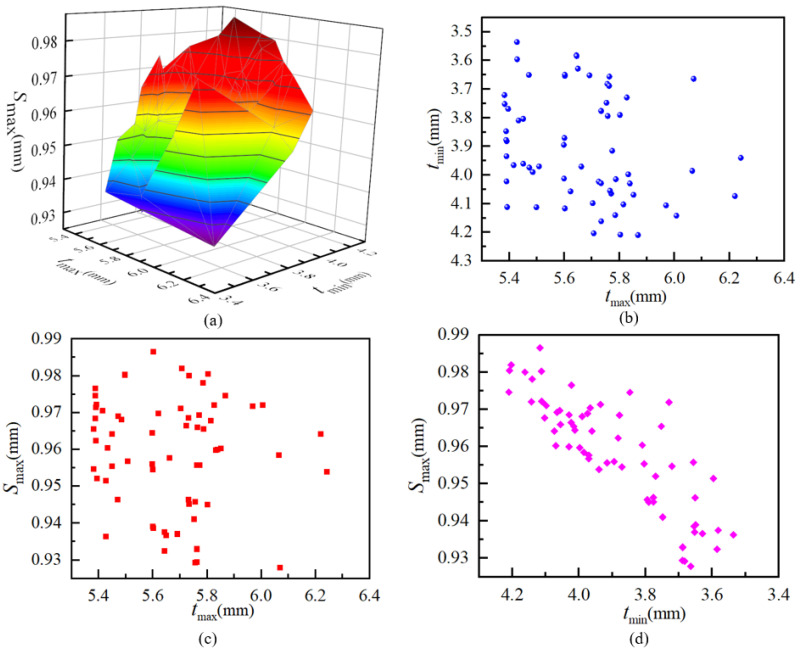
Pareto optimal solutions under constrained conditions: (**a**) 3D spatial surface of constrained three-objective Pareto optimal front, (**b**) projection of constrained Pareto solutions on the $t_{\max}$–$t_{\min}$ plane, (**c**) projection of constrained Pareto solutions on the $t_{\max}$–$S_{\max}$ plane, (**d**) projection of constrained Pareto solutions on the $t_{\min}$–$S_{\max}$.

Accordingly, constraint conditions were imposed on the objective functions to screen qualified optimal solutions, ensuring that the optimized results can be directly adopted for practical process design. With all other parameters kept unchanged, the upper bounds of $t_{\max}$ and $S_{\max}$ were set to 6.5 mm and 1.0 mm, respectively, while the lower bound of $t_{\min}$ was moderately relaxed to 3.5 mm. The results of constrained optimization are presented in Figure 20. It is observed that the optimized solution set converges uniformly to a complete and continuous irregular spatial surface, and all optimal solutions comply with process specifications. Compared with unconstrained optimization, the solution set of constrained optimization is confined within the feasible region, with its scale and coverage remarkably reduced. Only non-dominated solutions satisfying process requirements are retained. In addition, when the upper bound of $S_{\max}$ was set to 0.73 mm, no Pareto optimal solutions that meet the constraint requirements were generated after optimization.

### 5.3. Correlation Analysis

**Figure 21:**
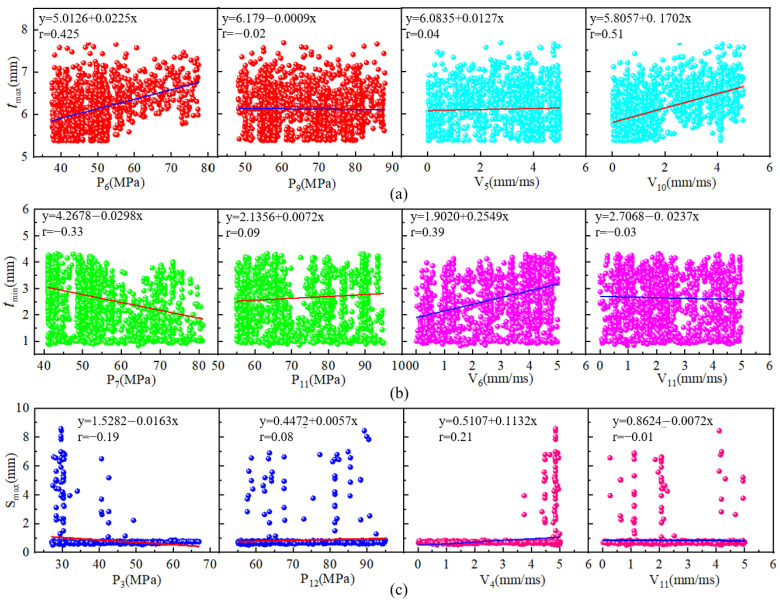
Correlation between partial design variables and optimization objectives: (**a**) correlation scatter plots of process variables versus maximum wall thickness $t_{\max}$, (**b**) correlation scatter plots of process variables versus minimum wall thickness $t_{\min}$, (**c**) correlation scatter plots of process variables versus maximum thickness deviation $S_{\max}$.

Table 3 lists the correlation coefficients between each design variable and optimization objective under unconstrained optimization conditions. As shown in the table, variables P_6_~P_8_ and V_8_~V_10_ are positively correlated with $t_{\max}$, while the remaining pressure and velocity variables exhibit no significant correlation with $t_{\max}$. P_6_ presents a negative correlation with $t_{\min}$, and V_4_–V_6_ show a positive correlation with $t_{\min}$. Most design variables exert weak or negligible effects on $t_{\min}$. In addition, individual pressure or velocity variables are weakly correlated or uncorrelated with $S_{\max}$. It can be observed from the stress–strain state model in Figure 6 that the intervals P_6_ to P_8_ correspond to the high-pressure bulging stage in the middle forming phase, where the tubular blank is subjected to multi-axial stress characterized by circumferential tension and insufficient axial material feeding. The internal pressure within this interval directly determines the magnitude of interfacial friction resistance between the die and tubular part. Higher internal pressure tends to hinder the axial material flow toward the central deformation zone. The circumferential tensile thinning at the central region cannot be compensated by axial incoming material, and redundant metal accumulates continuously at both ends of the tube, resulting in a remarkable increase in wall thickness at the tube ends. Hence, a strong correlation exists between internal pressure and wall thickness variation. In contrast, the forming stages corresponding to other low-pressure and pressure-holding control points feature weak frictional constraints or fully shaped blanks, which cannot induce large-scale metal accumulation. The feeding velocity can only slightly adjust the material feeding volume, and its regulation effect on wall thickness accumulation at tube ends is weaker than that of high internal pressure in the middle forming stage. Internal pressure is the dominant factor controlling tube wall thickness uniformity. Elevated internal pressure readily triggers wall thickening at tube ends and excessive thinning in the central section, which is consistent with forming theories and practical engineering laws. The influence of feeding velocity on wall thickness distribution differs across distinct forming stages. High-speed material feeding in the middle forming phase aggravates wall thinning, while reducing the feeding velocity at the late forming stage can effectively alleviate defects of uneven wall thickness. Meanwhile, $S_{\max}$ is predominantly governed by die cavity precision, initial blank dimensions, and the matching curve of pressure and axial feed (i.e., loading path), rather than a single process parameter. Accordingly, improving the die-fitting accuracy of tubular components requires priority optimization of the coordinated loading path, instead of simply adjusting internal pressure or feed velocity. The correlation distribution between partial variables and objective parameters is illustrated in Figure 21.

### 5.4. Discussion on Schemes

**Figure 22:**
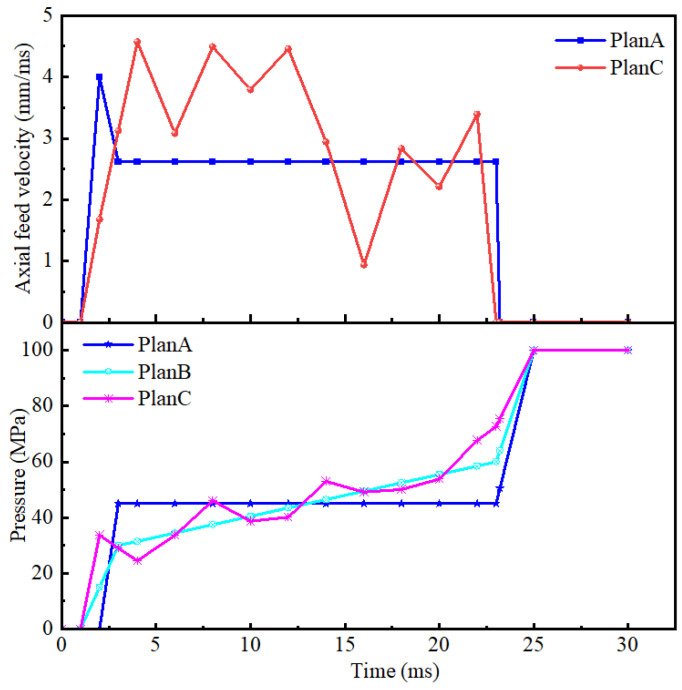
Pressure curve and feed velocity curve under three schemes.

To systematically verify the engineering effectiveness of the optimized results, three sets of hydroforming loading path schemes were designed in this study, and the detailed parameters are presented in Figure 22. Scheme A adopts a constant internal pressure combined with bidirectional axial feeding. Scheme B applies a loading mode where the internal pressure increases synchronously with axial feeding. Scheme C corresponds to the optimal loading strategy obtained via the present optimization. The left-side feeding curves remain identical across all three schemes, and the total simulation duration is set to 25 ms. Notably, the left-side feeding displacement of Scheme C is only 15.8 mm. This arrangement is designed to counteract interfacial friction resistance and achieve end sealing of the tube blank.

**Figure 23:**
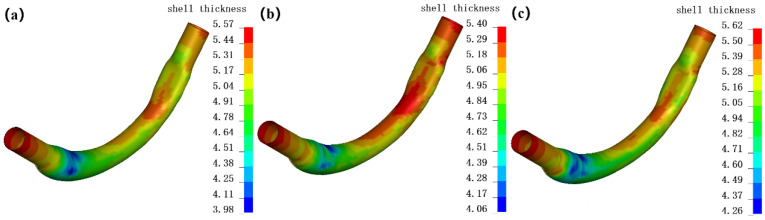
Simulation results using different process plans: (**a**) Plan A, (**b**) Plan B, (**c**) Plan C.

Figure 23 shows the wall thickness contours of the trailing arm under three loading plans. All three plans enable sound forming of the tubular component. The minimum wall thicknesses of Plan A, Plan B and Plan C are 3.98 mm, 4.06 mm and 4.26 mm, with wall thinning rates of 20.4%, 18.8% and 14.8% correspondingly. Their maximum wall thicknesses and thickening rates are 5.57 mm (11.4%), 5.40 mm (8%) and 5.62 mm (12.4%). Compared with the control plans, the optimized Plan C obviously alleviates wall thinning.

**Figure 24:**
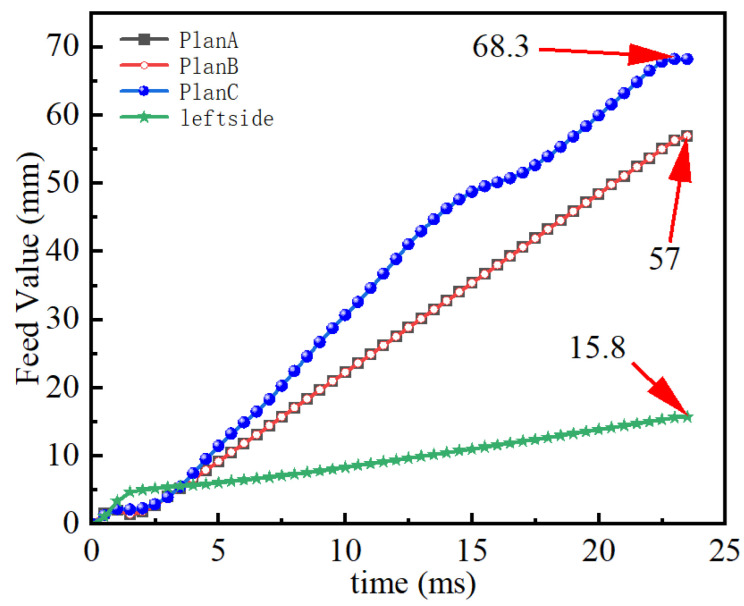
Simulation results using different process plans.

As can be seen from Figure 24, the maximum feeding displacement of Plan A and Plan B is 57 mm. For Plan C, this value rises by 11.3 mm to 68.3 mm, which greatly improves the forming quality.

## 6. Conclusions

This study establishes a multi-objective optimization model for hydroforming of automotive trailing arms. The optimization objectives include the maximum wall thickness, minimum wall thickness of the tube blank, and the clearance between the trailing arm and die, while the internal pressure and axial feeding velocity on the right side are defined as design variables. The loading path for hydroforming is optimized using the AMGA. The main conclusions are summarized as follows:

(1) Hydroforming of complex structural components is characterized by high-dimensional parameter coupling and mutual restriction among multiple objectives. The AMGA is adopted for simulation optimization in this work, which effectively addresses the drawbacks of traditional algorithms, such as slow convergence, insufficient solution diversity and susceptibility to local optima. This work fills the research gap of AMGA application in tube hydroforming and offers a valuable reference for process optimization of similar tubular parts.

(2) Compared with traditional empirically designed loading schedules, the optimized loading path significantly improves forming quality. The maximum wall thinning rate of the tubular part is reduced from 20.4% to 14.8%, while the wall thickening performance is well maintained. A full round of optimization calculation involving thousands of finite element solutions can generate a complete set of Pareto non-dominated solutions, providing diverse alternatives for hydroforming process planning and parameter matching.

(3) The hydroforming process of tubular parts is well suited to the collaborative loading mode with variable internal pressure and variable feeding velocity. Correlation analysis reveals that the axial feeding velocity acts as a core parameter governing the forming quality. A segmented dynamic feeding strategy effectively mitigates excessive thinning and cracking: low feed velocity at the initial and final stages suppresses wrinkling, while higher velocity is adopted in the middle forming stage.

(4) Physical experiments are carried out on a self-developed 2000-ton hydroforming apparatus to verify the engineering feasibility of the proposed optimization method. The experimental results show good agreement with the simulation predictions, with a maximum error of only 7.46%. The optimized loading path effectively alleviates the excessive wall thinning at the middle section and material accumulation at the ends induced by traditional processes. The proposed method is applicable to the forming of tubular parts with complex cross-sections, and provides reliable theoretical and technical support for intelligent manufacturing of profiled tubular components.

The coupled AMGA-LS-DYNA optimization framework established in this work exhibits excellent generalization performance. There is no need to reconstruct the core algorithm program; the framework can be extended to room-temperature and warm hydroforming working conditions of high-strength steel and aluminum alloys merely by replacing geometric models and calibrating corresponding constitutive parameters. At present, the proposed method is only validated via torsion beams made of SPHC steel. Multi-component and multi-material experiments will be carried out in subsequent research to quantify the cross-condition adaptability of the framework.

This optimization system can drastically cut physical trial batches and resolve the key drawbacks of traditional manual mold trials, including lengthy R&D cycles and unstable forming quality. Nevertheless, the numerical model still has several engineering limitations: it simplifies dies and end plugs as rigid bodies and employs a constant friction coefficient, neglecting die wear and time-varying interfacial friction behavior. The optimization objectives are only defined based on wall thickness and die-fitting gaps, without incorporating industrial metrics such as springback and forming energy consumption. In addition, only constraint boundaries are assigned to equipment parameters, while their collaborative multi-variable optimization is not performed simultaneously. Therefore, further improvements are required before full-scale industrial implementation.

For follow-up research, in-depth investigations will be conducted from three perspectives. First, simulations and physical experiments of tubular parts with various specifications and multiple metallic materials will be performed to systematically verify the universality of the framework. Second, ductile damage constitutive models and time-varying friction models will be introduced, and multi-objective evaluation indicators will be expanded to establish a simulation and optimization system that better conforms to real plastic flow behaviors. Third, an adaptive closed-loop loading control strategy will be developed by integrating online real-time detection technology to further enhance the precise process regulation capacity, facilitating the large-scale implementation of intelligent multi-objective optimization methods in the mass manufacturing of complex hollow tubular components.

## Figures and Tables

**Table 1:** Table of correspondence between element size and simulation results.

Element Size	Work Step	Element Number			Simulation Time		
		Die	Tube Blank	Total	Calculation Time	T_max_	T_min_
2 mm	Pre-bending	19,798	38,400	58,198	51 min 52 s	5.37	4.62
	Pre-forming	53,549	38,400	91,949	42 min 1 s	5.39	4.62
	Hydroforming	54,793	38,400	93,193	74 min 35 s	5.58	3.99
4 mm	Pre-bending	11,886	9600	21,486	12 min	5.49	4.62
	Pre-forming	13,404	9600	23,004	3 min 21 s	5.52	4.63
	Hydroforming	13,825	9600	23,425	6 min 19 s	5.60	3.97
6 mm	Pre-bending	10,338	4256	14,594	7 min 50 s	5.29	4.57
	Pre-forming	6208	4256	10,464	37 s	5.52	4.55
	Hydroforming	6281	4256	10,537	1 min 6 s	5.57	3.98

**Table 2:** Statistics of simulation prediction deviation at each measuring point of transverse section.

Measuring Point	Section A	Section B	Section C	Section D	Section E
T1	−2.95%	−4.6%	−3.86%	−3.08%	−6.97%
T2	0.24%	−4.1%	−4.82%	−2.12%	−6.45%
T3	0.16%	1.41%	−3.08%	0.46%	−3.1%
T4	0.4%	−2.06%	−5.23%	0.21%	−2.78%
T5	−3.83%	−7.16%	−4.49%	−1.18%	−6.77%
T6	−2.72%	−3.99%	−5.55%	−4.92%	−2.95%
T7	−4.55%	−7.31%	−6.91%	−6.57%	−3.14%
T8	−3.07%	−7.46%	−4.58%	2.23%	−2.3%

**Table 3:** The correlation coefficients between design variables and optimization objectives.

**Variables**	**P_1_**	**P_2_**	**P_3_**	**P_4_**	**P_5_**	**P_6_**	**P_7_**	**P_8_**	**P_9_**	**P_10_**	**P_11_**	**P_12_**
$t_{max}$	0.16	0.28	0.18	0.25	0.09	0.43	0.40	0.32	−0.02	0.19	0.19	0.18
$t_{min}$	−0.28	−0.002	−0.18	0.04	−0.22	−0.27	−0.33	−0.14	−0.17	−0.11	0.09	−0.13
$S_{max}$	−0.11	0.02	−0.19	0.02	−0.13	−0.04	−0.13	−0.10	−0.17	0.01	0.04	0.08
Variables	V_1_	V_2_	V_3_	V_4_	V_5_	V_6_	V_7_	V_8_	V_9_	V_10_	V_11_	
$t_{max}$	0.29	0.24	0.22	0.10	0.04	0.06	0.18	0.33	0.43	0.51	0.19	
$t_{min}$	0.16	0.17	0.27	0.38	0.30	0.39	0.26	0.27	0.13	−0.03	−0.03	
$S_{max}$	0.05	0.19	0.17	0.21	0.18	0.16	0.12	0.14	0.17	0.02	−0.01	

## Data Availability

The authors confirm that the supporting data for this study’s conclusion are included in the manuscript. Raw simulation and experimental data are available from the corresponding author upon reasonable request.
